# Paraquat-Mediated Oxidative Stress in *Anopheles gambiae* Mosquitoes Is Regulated by An Endoplasmic Reticulum (ER) Stress Response

**DOI:** 10.3390/proteomes6040047

**Published:** 2018-11-12

**Authors:** Brian B. Tarimo, Henry Chun Hin Law, Dingyin Tao, Rebecca Pastrana-Mena, Stefan M. Kanzok, Joram J. Buza, Rhoel R. Dinglasan

**Affiliations:** 1 Department of Health and Biomedical Sciences, Nelson Mandela-African Institution of Science and Technology, Tengeru, Arusha 23302, Tanzania; btarimo@ihi.or.tz (B.B.T.); joram.buza@nm-aist.ac.tz (J.J.B.); 2 W. Harry Feinstone Department of Molecular Microbiology & Immunology & the Malaria Research Institute, Johns Hopkins Bloomberg School of Public Health, Baltimore, MD 21205, USA; dingyin.tao@nih.gov (D.T.); rebecca.pastrana.mena@gmail.com (R.P.-M.); 3 Department of Environmental Health & Ecological Sciences, Ifakara Health Institute, Dar es Salaam 14112, Tanzania; 4 Emerging Pathogens Institute, Department of Infectious Diseases & Immunology, College of Veterinary Medicine, University of Florida, Gainesville, FL 32611, USA; henrylawch@connect.hku.hk; 5 Department of Biology, Loyola University Chicago, Chicago, IL 60660, USA; skanzok@luc.edu

**Keywords:** malaria, *Anopheles gambiae*, oxidative stress, endoplasmic reticulum stress, paraquat, transmission-blocking

## Abstract

Paraquat is a potent superoxide (O_2_^−^)-inducing agent that is capable of inducing an oxidative imbalance in the mosquito midgut. This oxidative imbalance can super-stress the malaria parasite, leading to arrested development in the mosquito midgut and reduced transmission. While several studies have explored the effect of paraquat on malaria parasites, a fundamental understanding of the mosquito response to this compound remains unknown. Here, we quantified the mosquito midgut proteomic response to a paraquat-laced sugar meal, and found that *An. gambiae* midguts were enriched in proteins that are indicative of cells under endoplasmic reticulum (ER) stress. We also carried out qRT-PCR analyses for nine prominent thioredoxin (Trx) and glutathione (GSH)-dependent genes in mosquito midguts post *P. falciparum* blood meal ingestion to evaluate the concordance between transcripts and proteins under different oxidative stress conditions. Our data revealed an absence of significant upregulation in the Trx and GSH-dependent genes following infected blood meal ingestion. These data suggest that the intrinsic tolerance of the mosquito midgut to paraquat-mediated oxidative stress is through an ER stress response. These data indicate that mosquitoes have at least two divergent pathways of managing the oxidative stress that is induced by exogenous compounds, and outline the potential application of paraquat-like drugs to act selectively against malaria parasite development in mosquito midguts, thereby blocking mosquito-to-human transmission.

## 1. Introduction

Malaria caused by the protozoan parasite *Plasmodium* remains a major global public health problem, despite extensive investment in the control and elimination of this disease. A major gap in knowledge in this field is our understanding of the various processes that occur during parasite transmission through its insect vector, the *Anopheles* mosquito. Following *Plasmodium*-infected blood meal ingestion by *Anopheles* mosquitoes, the parasite undergoes an intricate developmental stage in the mosquito. *Plasmodium* gametocytes in the ingested blood fuse and undergo sexual reproduction to form a zygote that shortly thereafter transforms into a motile ookinete. The ookinete then leaves the blood bolus to invade and traverse the mosquito midgut epithelium to its basal lamina side, where it differentiates into an oocyst. The oocyst grows in size and undergoes sporogony to produce thousands of sporozoites. Once the oocyst matures, it ruptures and releases the sporozoites into the hemocoel, where they are transported passively to the salivary glands. A mosquito with a salivary gland infection with sporozoites reestablishes the cycle in the vertebrate host by transmitting the sporozoites through its saliva as it blood feeds [1].

The success of *Plasmodium* is in part because the parasite has evolved a balance with its insect host. Disrupting this balance could lead to new interventions that can reduce, if not completely block, malaria transmission. *Plasmodium* parasite development in *Anopheles* mosquitoes is associated with excessive amounts of reactive oxygen species (ROS) and reactive nitrogen species (RNS) from several sources. These sources include vertebrate immune factors that are present in the ingested blood [2,3], the digestion of hemoglobin [4,5], and the mosquito’s innate immunity due to invasion of its midgut epithelial cells by the parasite [6,7,8]. Oxidative stress could be fatal to cells if not immediately dealt with due to its ability to cause damage to cellular macromolecules such as proteins, cell membranes, and nucleic acids [9].

**Figure 1:**
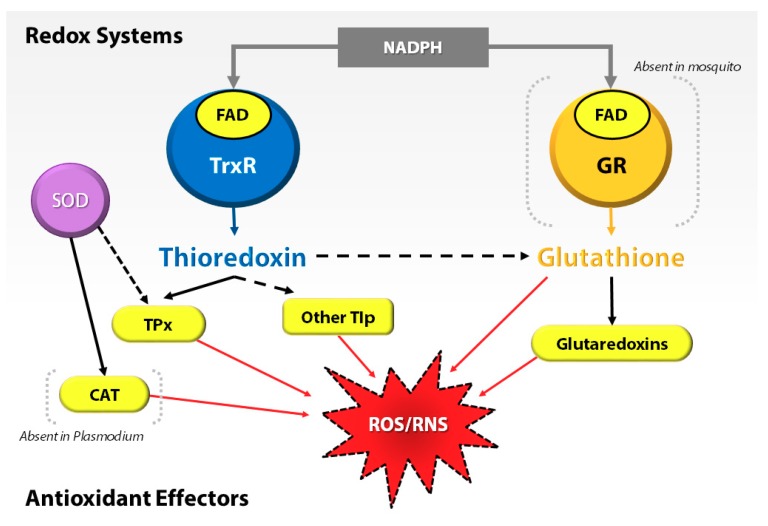
Interplay of redox systems in *Plasmodium* and mosquito. ROS/RNS = reactive oxygen species/reactive nitrogen species, GR = glutathione reductase, FAD = flavin adenosine dinucleotide, NADPH = reduced nicotinamide dinucleotide phosphate, TrxR = thioredoxin reductase, SOD = superoxide dismutase, TPx = thioredoxin peroxidase, CAT = Catalase, and Tlp = thioredoxin-like proteins.

At the cellular level, most organisms depend on the thioredoxin (Trx) and glutathione (GSH) systems as prominent lines of defense against oxidative stress. For example, in *Plasmodium*, the absence or deficiency of an antioxidant gene, e.g., glutathione reductase (GR), severely affects the development of the parasite in the insect host [10,11]. Interestingly, *Anopheles* mosquitoes, similar to other dipterans, lack the GR and compensate by utilizing the Trx system to recycle oxidized glutathione (GSSG) to GSH [12] (Figure 1). This emphasizes the importance of the Trx system in oxidative stress regulation in *Anopheles* mosquitoes. Thus, they regulate the proteins of the Trx system to protect midgut epithelial cells against oxidative stress, specifically when associated with infection by the malaria parasite [13,14].

Existing data on redox homeostasis in the *Anopheles* mosquito midgut due to *Plasmodium* ookinete invasion has been largely generated using the *P. berghei*/*An. stephensi*/*An. gambiae* parasite–vector model, which is not a natural parasite–vector system of malaria transmission. The natural, co-evolved, parasite–vector system that is responsible for malaria morbidity and mortality in sub-Saharan Africa is *P. falciparum*/*An. gambiae*. Exposure to the ROS inducer paraquat (Pqt; 1,1′-dimethyl-4,4′-bipyridylium di-chloride) upregulates antioxidant responses in *P. berghei ookinete* in vitro [15]. Considering its antiparasitic activity, we propose that Pqt-induced oxidative stress can be exploited as a transmission-blocking strategy against human malaria parasites. The concept is straightforward: high concentrations of ROS-inducing compounds such as Pqt, which are taken up along with a *Plasmodium*-infected blood meal, would prove to be deleterious to the parasites, but still be tolerable by the mosquito. In such a scenario, the goal is to prevent mosquitoes from infection by malaria parasites rather than reduce vector populations. Although the *Anopheles* midgut antioxidant response following ingestion of an infected/uninfected blood meal has been extensively studied [4,5,8,14,15,16,17,18,19], the exclusive midgut response to an ROS-inducing compound in the absence of a blood meal remains unknown. We hypothesize that the mosquito vector can mitigate the extra ROS produced following the ingestion of Pqt, permitting selective toxicity against malaria parasites during the first 24 h following the ingestion of infected blood. We further hypothesize that tolerance to high concentrations of Pqt in the absence of a blood meal would suggest that the mosquito has in place a repertoire of cellular responses that permit the regulation of ROS above and beyond those induced by both an infected or non-infected blood meal itself [4,5,8,14,15,16,17,18,19]. To explore the feasibility of this proposed transmission-blocking strategy, we used a quantitative proteomic approach to profile the organ (midgut)-level response of *An. gambiae* mosquitoes to Pqt-induced oxidative stress. To evaluate the concordance between transcripts and proteins under different oxidative conditions, we measured the expression profile of Trx and GSH-dependent genes in *An. gambiae* midguts 24-h post *P. falciparum* blood meal ingestion.

## 2. Materials and Methods

### 2.1. Mosquito Rearing, Experimental Treatments, and ROS Induction Assays

*An. gambiae* (KEELE strain) mosquitoes were used for all of the experimental treatments. These mosquitoes were maintained in an insectary at the Johns Hopkins Malaria Research Institute (JHMRI), kept at 26 °C and 70% humidity with 12-h light and dark cycles, and supplemented with 10% sucrose solution.

ROS induction (oxidative stress) assays were performed using mosquito-feeding experiments. Considering the difficulties in performing LC-MS/MS analyses on digested blood, our methodological approach was limited to characterizing the midgut proteomic response following the direct ingestion of Pqt in the absence of a blood meal. Membrane feeding assays (MFA) used 50–75 *An. gambiae* female mosquitoes (four to seven days old) that were pre-starved for >18 h. An ATP-saline solution [150 mM of NaCl, 10 mM of NaHCO3 pH 7.0 [20], and 1 mM of ATP added as a phagostimulant [21,22,23]] containing the following treatments: 10% sucrose solution (control group) or 1 mM of Pqt (treatment group; Sigma-Aldrich, St. Louis, MO, USA), based from a previous similar study on *An. stephensi* mosquitoes [23], was prepared to a 2× concentration. To track uptake, an equivalent volume of colored water (artificial red food color) was added to the experimental groups, and then delivered directly into glass, water-jacketed membrane feeders warmed to 37 °C. Mosquitoes were allowed to feed for 30–45 min. The fed mosquitoes were kept at 26 °C and 70% humidity for 8 h and maintained on sugar (10% sucrose) and water. We selected the eight-hour time frame because of its reasonable relevance to when parasite developmental stages (zygote-to-ookinete transition stages) are present in the midgut following the ingestion of an infectious blood meal. The colored water in the treatments aided the selection of only those mosquitoes that fed on the solution. The artificial food color that was used did not contain any ingredients known to either favor or hinder the production of ROS/RNS. The midguts were dissected from 50 fully fed mosquitoes per experimental group, and transferred into 200 μL 1× Phosphate-buffered saline (PBS) on ice. All of the 1× PBS was removed, and the samples were stored at −80 °C until further liquid chromatography-tandem mass spectrometry (LC-MS/MS) analysis. These experiments were replicated three times using independent biological cohorts of mosquitoes to ensure reproducibility.

Standard membrane feeding assays (SMFA) used pre-starved 50–75 *An. gambiae* female mosquitoes (four to seven days old) per treatment group. *P. falciparum* (NF54) gametocyte cultures (15–18 days post-initiation) were pelleted and diluted to 1.0% gametocytemia with human blood (Interstate Blood Bank, Memphis, TN) that had been washed with RPMI 1640 (Thermo Fisher Scientific) and brought up to 50% hematocrit with normal AB serum. Gametocytemic blood was kept at 37 °C until feeding. During feeding, 200 μL of gametocytemic blood (experimental treatment), human blood at 50% hematocrit (blood control treatment), and 10% sucrose solution (sugar control treatment) were delivered directly into glass, water-jacketed membrane feeders warmed to 37 °C. Mosquitoes were allowed to feed for 30–45 min. After blood feeding, non-blood fed mosquitoes were removed from each treatment group, and the fed mosquitoes were maintained on sugar and water at 26 °C and 70% humidity (to assure survival and prevent desiccation) for 24 h prior to midgut dissections. Midguts were dissected into TRIzol reagent (Thermo Fisher Scientific, Waltham, MA, USA) for total RNA extraction. The human blood that was used in our experiments was procured through an established blood bank with ethically approved collection practices. These experiments were replicated three times using independent biological cohorts of mosquitoes.

### 2.2. Extraction, Solubilization, and Digestion of Proteins

Prior to LC-MS/MS analysis, experimental groups (50 midguts/sample) were processed as follows. Total protein lysate was prepared by lysing the midgut samples with 45 μL of SDST-lysis buffer (4% Sodium dodecyl sulfate [SDS] (*w*/*v*), 100 mM of Tris/HCl, 0.1 M of dl-dithiothreitol, pH 7.6) and boiled at 95 °C for 5 min. An aliquot of the protein lysate (30 μL) was used for protein digestion according to the filter-aided sample preparation (FASP) protocol [24] using a 10-kDa molecular weight cutoff filter [25,26] (EMD Millipore, Billerica, MA, USA). Acidified tryptic peptides from following FASP treatment were desalted using an HPLC C18 column on an Agilent 1200 HPLC system (Agilent Technologies, Santa Clara, CA, USA) [25,26]. Concentration of the peptides following FASP was estimated using protein digest standards whose concentrations, as determined by bicinchoninic acid assay (BCA), were known [25,26].

### 2.3. Online 2D LC-MS/MS Analysis

Peptide products desalted and digested by the FASP protocol were dissolved in loading buffer (97.9% water, 2% CAN, and 0.1% formic acid (FA)), and ~20 μg was injected to an online two-dimensional (2D) HPLC-MS/MS system, using the exact method as described previously and briefly detailed below [25,26,27]. The online 2D HPLC-MS-MS system was constructed by integrating one strong cation exchange (SCX) column (150 μm i.d. × 2 cm length PolySULFOETHYL A^TM^, 5 μm 300 Å, PolyLC INC) into an Agilent LC-MS system comprised of a 1200 LC system coupled to a 6520 QTOF via an HPLC Chip Cube interface. For the online SCX fractionation, in the first dimension, peptides were loaded into the SCX column at 1.8 μL/min, and the peptides were eluted using the autosampler by injecting 6 μl of each increasing salt concentration (0, 15 mM, 30 mM, 45 mM, 60 mM, 120 mM, 160 mM, and 300 mM of NaCl in 2% ACN/0.1% FA) followed by one injection of 500 mM of NaCl in 2% ACN/0.1% FA to wash the column. The salt elution was captured by a C18 enrichment column integrated into the Agilent Polaris-HR-Chip-3C18 chip (360 nL, 180 Å C18 trap with a 75 μm i.d., 150 mm length, 180 Å C18 analytical column). In the second dimension, with the valve switched and the Reversed-phase liquid chromatography (RPLC) gradient started, the peptides were eluted from the enrichment column and separated by a C18 analytical column. The elution of peptides from the analytical column was performed using a gradient starting at 97% A (A: 99.9% water, 0.1% FA) at 300 nL/min. The mobile phase was 3–10% B (B: 90% ACN, 9.9% water, 0.1% FA) for four minutes, 10–35% B for 56 minutes, 35–99% B for two minutes, and maintained at 99% B for six minutes, followed by re-equilibration of the column with 3% B for 10 min. Data-dependent (autoMS2) MS acquisition was performed by an Agilent 6520 QTOF (Agilent Technologies, Wilmington, DE, USA) at 2 GHz. Precursor MS spectra were acquired from *m*/*z* 315 to 1700, and the top four peaks were selected for MS/MS analysis. Product scans were acquired from *m*/*z* 50 to 1700 at a scan rate of 1.5 spectra per second. A medium isolation width (~4 amu) was used, and a collision energy of slope 3.6 V/100 Da with a 2.9 V offset was applied for fragmentation. A dynamic exclusion list was applied with precursors excluded for 0.50 min after two MS/MS spectra were acquired.

### 2.4. Database Searching and Label-Free Quantification Analysis

All of the LC-MS/MS raw data were converted to Mascot generic format (.mgf) by Agilent MassHunter Qualitative Analysis B.04.00 (Agilent Technologies, Wilmington, DE, USA). The data acquired was used to search the VectorBase *Anopheles gambiae* protein FASTA sequence database (VectorBase, http://www.vectorbase.org, *Anopheles gambiae* PEST, AgamP4.2.) for peptide sequence alignments. The search engine that was used for the search was MASCOT version 2.5 (Matrix Science, Boston, MA, USA) with the following parameters: precursor ion mass tolerance of 50 ppm, fragment ion mass tolerance of 0.2 Da, carbamidomethylation of cysteine, and oxidation of methionine residues set as fixed and variable modifications, respectively. Peptides were searched using fully tryptic cleavage constraints, and up to two internal cleavage sites were allowed for tryptic digestion. The MASCOT search results were exported as .DAT format and then imported into the Scaffold software (version 4.4.5, Proteome Software, Portland, OR, USA) for curation, label-free quantification, analysis, and visualization. Overall, protein false discovery rates of less than 1% and peptide false discovery rates of less than 1% were obtained with Scaffold filters, and each protein had ≥2 unique peptides. Identified proteins were clustered to remove redundancy. Proteins were clustered together if there was a peptide identification shared between them, because this indicates substantial sequence similarity, and the protein with the greatest number of peptides identified was considered the unique protein identification from that group. The data analysis pipeline meets all of the MIAPE standards [28], and the detailed peptide data can be found in the Supporting Information (Table S1). The mass spectrometry proteomics data have been deposited to the ProteomeXchange Consortium via the PRIDE partner repository with the dataset identifier PXD008251 [29].

### 2.5. qRT-PCR

Total RNA was extracted from samples of in vivo studies with SMFA using TRIzol reagent () according to the manufacturer’s protocol. Extracted RNA was checked for purity and concentration using the Nanodrop 2000 UV-Vis spectrophotometer (Thermo Fisher Scientific, Waltham, MA, USA). cDNAs was synthesized using the RevertAid First Strand cDNA Synthesis Kit (Thermo Fisher Scientific, Waltham, MA, USA). Quantitative RT-PCR was performed using SYBR Green Master Mix (Applied Biosystems, Carlsbad, CA, USA) on a StepOnePlus Real-Time PCR System (Applied Biosystems, Foster City, CA, USA).

Relative transcript levels of the thioredoxin system (Trx-1, Trx-2, TrxR, Tpx-1, and PrxV) and glutathione system (Grx-1, GSTD1, GPx, and GS,) were determined using gene-specific primers and cycling conditions as per manufacturer’s protocol. Expression levels were calculated using the 2^−∆∆Ct^ method [30] relative to the *Anopheles gambiae* ribosomal protein RpL32 (AgRpL32; AGAP002122) gene, which was amplified using AgRpL32 F 5′- GCCGAAGATTGTGAAGAAGC-3′ and AgRpL32 R5′- GCACCCGATTGTCAATACCT-3′. All of the qRT-PCR reactions were done in triplicate. Specific primer sequences of the transcripts can be found in (Table S2) in the Supplemental Material.

### 2.6. Statistical Analyses

The Student’s *t*-test comparing quantifiable spectral values between treatment groups was used to identify the differentially expressed proteins. For qRT-PCR analyses, comparisons of expression levels of targeted transcripts relative to each other were carried out using multiple Student’s *t*-tests followed by a Holm–Šidak correction of t-scores to adjust for multiple tests. All of the statistical analyses utilized the software GraphPad Prism (version 6.0e, GraphPad Software, La Jolla, CA, USA). The *p*-values of <0.05 were considered statistically significant. All of the experimental reactions used at least three independent biological replicate samples.

## 3. Results

The presence of a blood meal itself prevents the specific, accurate mass spectrometry-based proteomic analysis of the midgut response to Pqt alone from a Pqt-laced blood meal. Given this limitation, we sought to identify the midgut’s response to Pqt alone, considering that the blood meal induces a specific antioxidant response by the mosquito midgut. The assumption is that the identified processes would be in addition to those predicted to be mounted by the midgut in order to maintain redox homeostasis during blood feeding and digestion. With this in mind, we used a label-free quantitative proteomic approach to determine the midgut-level regulation of the response to Pqt-induced oxidative stress by *An. gambiae* mosquitoes; thereby complementing several studies that have explored the effect of Pqt on malaria parasites.

### 3.1. Global Proteomic Profiles of Midgut Epithelial Cells under Pqt-Induced Oxidative Stress Are Largely Conserved

**Figure 2:**
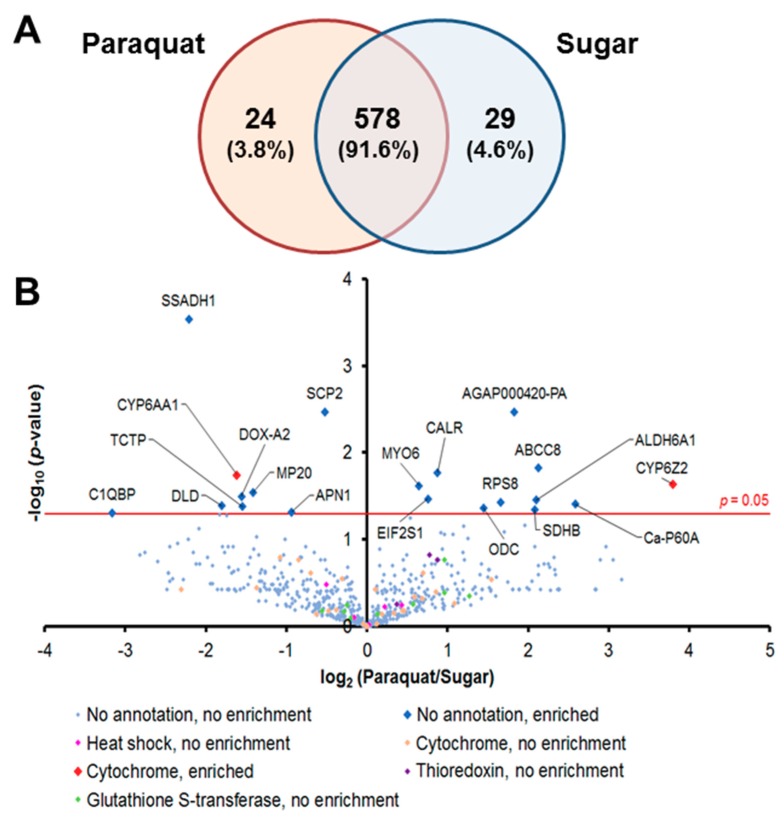
Comparative label-free quantitative proteomic analyses of the *An. gambiae* midgut responses to the ROS-inducer paraquat (Pqt). (**A**) Global distribution of proteins in midguts under Pqt and sugar solution treatment. Midgut lysates from female *Anopheles gambiae* mosquito midguts treated with 1-mM concentration of Pqt and sugar (10% sucrose) solution were subjected to a LC-MS/MS analysis to identify expressed proteins. Of the 631 proteins quantified, 3.8% were specific to Pqt-treated midguts, 4.6% were partitioned to sugar-treated midguts, and 91.6% of the total proteins were conserved in both the Pqt and sugar-treated midguts. (**B**) Protein identification comparisons between treatment groups in *An. gambiae* midguts. Midgut lysates from female *An. gambiae* mosquito midguts treated with Pqt were subjected to LC-MS/MS analysis to identify expressed proteins. Volcano plots of quantifiable protein comparisons in Pqt vs. sugar (10% sucrose) solution-treated midguts. Significant fold change was calculated with Student’s *t*-test with a *p*-value ≤ 0.05. The red line indicates a *p*-value = 0.05. The annotation of significantly enriched proteins is shown.

We captured the proteomic profiles of *An. gambiae* midguts dissected 8 h after ingestion of a 1-mM Pqt-laced sugar meal. This concentration of Pqt was found to induce oxidative stress without noticeable fatal damage to the midgut epithelial cells, which was evident in the absence of loss of tissue structure (data not shown). We analyzed the global proteomic profile in midgut epithelial cells treated with Pqt (experimental group) and sugar (control group). We identified a total of 631 quantifiable proteins by label-free techniques with a protein false discovery rate of <1% and normalization based on area under the curve (Table S3). We observed that 578 proteins (91.6%) were shared between Pqt and sugar-treated midguts, 24 (3.8%) were found only in Pqt-treated midguts, and 29 (4.6%) were found only in sugar-treated midguts (Figure 2A). Antioxidant groups of proteins were organized from the 631 identified proteins, and this included heat shock proteins (HSP), cytochrome P450s (CYP), Trx-dependent proteins, and GSH-dependent proteins (Table S3). The quantitative proteomic profiles of the Pqt and sugar treatment groups identified 20 out of the total of 631 (0.031%) proteins that were differentially expressed between the groups based on spectral counts (*p* ≤ 0.05; Student’s *t*-test) (Figure 2B and Table S3). We found 11 out of the 20 (55%) proteins enriched (highly expressed) in Pqt-treated midguts. Annotated functions revealed that seven (63.6%) of these proteins are involved in the endoplasmic reticulum (ER) stress response or cellular detoxification machinery (Table 1).

### 3.2. Antioxidant Proteins Are Not Involved in the Regulation of Pqt-Induced Oxidative Stress in An. Gambiae Midguts

A detailed examination of the proteins identified as antioxidants that were previously described (in Section 3.1) was performed. Cytochrome P450 6Z2 (CYP6Z2; AGAP008212) was found to be enriched 13.86-fold (*p*-value = 0.023) in Pqt-treated midguts, and was the only CYP450 protein whose enrichment was statistically significant. We observed that CYP9J4 (AGAP012292) and CYP4H24 (AGAP013490) were also enriched (>1.5 fold), but their enrichment was not deemed to be statistically significant (Table S3). Although thioredoxin reductase (TrxR; AGAP000565) and thioredoxin peroxidase 4 (TPx-4; AGAP011824) were found to be enriched (>1.5-fold) in the Pqt-treated mosquitoes, their enrichment was not deemed statistically significant (Table S3). Glutathione S-transferase epsilon class 3 (GSTE3 AGAP009197), glutathione S-transferase theta class 1 (GSTT1; AGAP000761), and glutathione S-transferase delta class 11 (GSTD11; AGAP004378) were the only GSH-dependent proteins that were found to be enriched (>1.5-fold), but their enrichment was also not deemed to be statistically significant (Table S3). We did not identify any significant enrichment in any of the identified HSPs.

### 3.3. Evidence of an Endoplasmic Reticulum (ER) Stress Response in Pqt-Treated Midguts

We noted that ER stress-regulating proteins were enriched in Pqt-treated midguts relative to sugar-treated midguts. This includes calreticulin (CRT; *p*-value = 0.017), eukaryotic translation initiation factor 2 subunit alpha (EIF2S1; *p*-value = 0.034) and calcium-transporting ATPase sarcoplasmic/endoplasmic reticulum type (Ca-P60A; *p*-value = 0.039), which had fold changes of 1.84, 1.69, and 5.99-fold, respectively (Figure 2B; Table 1).

### 3.4. Proteins Involved in the Detoxification Process Are Enriched in Pqt-Treated Midguts

ATP-binding cassette transporter family C member 8 (ABCC8; AGAP008437) was found enriched by 4.37-fold (*p*-value = 0.015) in the Pqt-treated midguts (Table 1). ATP-binding cassette (ABC) transporters belong to a superfamily of transport system members that efflux drugs, toxic, endo, and xenobiotic compounds from cells [45,46]. Ornithine decarboxylase (ODC) was another detoxification protein that was found to be enriched in the Pqt-treated midguts (2.72-fold, *p*-value = 0.043) (Table 1). OCD catalyses the first-rate limiting step in polyamine synthesis, and is upregulated in response to stress.

We also found that mitochondrial methylmalonate-semialdehyde dehydrogenase ALDH6A1 (AGAP002499) was enriched by 4.28-fold (*p*-value = 0.035) in the Pqt-treated midguts (Table 1). A contig from the male accessory gland/testis vas deferens (MAG/TVD) of tick, *Dermacentor variabilis*, was identified through alignment to be ALDH6A1, and known to protect against environmental stress [32]. Succinate dehydrogenase (ubiquinone) iron–sulfur subunit (SDHB; AGAP0031) was enriched in the Pqt-treated midguts (4.23-fold, *p*-value = 0.045) (Table 1). SDHB is one of the four subunits of the mitochondrial succinate dehydrogenase complex (SDH), which is a key enzyme that links the tricarboxylic acid cycle (TCA) and electron transport chain (ETC) [47,48]. SDHB transfers electrons from flavin adenosine dinucleotide (FADH_2_) to ubiquinone (CoQ) in the inner mitochondrial membrane.

### 3.5. P. Falciparum Ookinete Invasion of An. Gambiae Midguts Does Not Upregulate Trx and GSH-Dependent Genes

The *P. berghei* ookinete invasion of *Anopheles* mosquitoes is accompanied by an increased production of ROS/RNS [6,7,8]. Since the proteomic profiling suggested that several of the Trx and GSH-dependent proteins were not upregulated following Pqt treatment, we carried out qRT-PCR analysis to look closely at the regulation of nine Trx and GSH- dependent genes in *An. gambiae* midguts following the ingestion of *P. falciparum* infected blood meal (Table 2). The Trx and GSH-dependent transcripts that were chosen were identified in various studies to be involved in the regulation of oxidative stress or in the detoxification of xenobiotic compounds. Multiple statistical analyses were carried out to identify the transcripts that were significantly upregulated in Trx and GSH pathways 24-h post-infected blood meal ingestion (Table S4). We found no significant upregulation of any of the Trx and GSH-dependent transcripts that were investigated (Table 2).

## 4. Discussion

We expected that Trx and GSH-dependent proteins would be significantly enriched in mosquito midguts following Pqt treatment. However, we did not observe any significant enrichment in the antioxidant proteins that were identified in our proteomic data. The absence of the enrichment of these proteins might be due to an early read-out time after Pqt treatment (8 h), a low Pqt concentration used (1 mM), or the delivery route of Pqt in an ATP solution through membrane feeding. The 8-h period was selected, as it is critically relevant in the context of gamete-to-ookinete transition, as well as in ookinete maturation. Furthermore, our intention was to determine whether the Pqt concentration of 1 mM elicits a Trx and GSH-dependent protein response in the mosquito. In a study on acute Pqt toxicity in *Drosophila melanogaster*, concentrations between 10–40 mM were used with the exposure time of 24 h resulting in significant elevation in oxidative stress biomarkers and antioxidant enzymes [55]. The antioxidant enzymes that were investigated were not Trx or GSH-dependent apart from GSTs. However, it clearly shows that the concentration of Pqt in our experiments might have been too low, and the exposure time too short, which could explain the lack of enrichment of Trx and GSH-dependent antioxidant proteins.

Considering the difficulty in conducting LC-MS/MS analyses of a blood-fed midgut at 24 h [56], we utilized qRT-PCR analyses to perform a sensitive, separate assessment of the regulation of nine Trx and GSH-dependent genes. *P. berghei* ookinetes appear to damage the mosquito midgut epithelium during midgut invasion and traversal, more so than *P. falciparum* ookinetes, due to the destructive nature of its invasion process. The invasion of epithelial cells induces the expression of nitric oxide synthase (NOS); this catalyses the formation of nitric oxide (NO) [4,6,7,8], which is a highly reactive RNS [57]. As Trx and GSH pathways are the primary cellular antioxidant and antinitrosative defence, we expected to observe an increase in the expression of Trx and GSH-dependent genes following *Plasmodium*-infected blood meal ingestion. The absence of significant upregulation of the investigated antioxidant genes could indicate that *P. falciparum* ookinete invasion of the *Anopheles* midgut does not cause significant oxidative stress when compared to that observed for *P. berghei*. This could be due to either/or a combination of the following reasons. First, *P. falciparum* has co-evolved with *An. gambiae*, and causes less destruction of midgut epithelial cells during midgut invasion compared to *P. berghei* ookinetes [6,7], because the total number of ookinetes that leave the blood bolus and invade the midgut epithelium is less in *P. falciparum* compared to *P. berghei* evident in the reported divergent oocyst intensities for the two *Plasmodium* species [58,59,60]. Second, the antioxidant response in the midgut is already activated due to the blood meal content, and the presence of *P. falciparum* doesn’t result in any significant [61] change.

Additional experiments are required at earlier time points, between 0–20 h, following *P. falciparum*-infected blood meal ingestion to detect potential variations in the expression of Trx and GSH-dependent genes. Furthermore, different tissues of the mosquito midgut experience increased levels of oxidative stress at different time points, depending on the development stage reached by the *Plasmodium* parasite. From blood meal ingestion to 15 h, increased levels of ROS/RNS are present in the blood bolus due to vertebrate immune factors and the digestion of hemoglobin in the blood meal [2,3,4,5]. Between 15–24 h, increased levels of ROS/RNS are in the midgut epithelial cells due to the initiation of the asynchronous invasion of *Plasmodium* ookinetes [6,7,8], and because blood digestion is reaching its midway point toward completion (~48 h post-blood feeding) [62]. Although *P. falciparum* ookinete invasion of the midgut is at its maximum at 24 h [62], it is entirely plausible that the levels of ROS/RNS in the midgut have already been reduced at this time point due to either advanced progress in the digestion of hemoglobin and/or the small number of *P. falciparum* ookinetes involved in the invasion process.

The absence of a significant enrichment in antioxidant proteins following the ingestion of a Pqt-laced sugar meal prompted us to closely examine the identified enriched proteins in the context of oxidative stress regulation. The mosquito midgut epithelial cells response to Pqt appears to be mediated primarily through the midgut ER–stress pathway, which was indicated by our finding that detoxification and ER stress-related proteins were enriched in the Pqt-treated midguts. Oxidative stress is known to increase the amount of misfolded or unfolded protein in a cell. The unfolded protein response (UPR) is a cellular surveillance mechanism that identifies misfolded proteins in the ER, and then either repairs them or redirects those that are misfolded beyond repair to the degradative pathway [62]. Therefore, the UPR coordinates the ER protein-folding demand and capacity with regards to the homeostatic status of a cell.

Pqt at the 1-mM concentration that was used in our experiments resulted in the misfolding and unfolding of proteins, prompting their immediate repair. The enriched levels of CRT, EIF2S1, and Ca-P60A are UPR-induced due to the ER stress in midgut epithelial cells caused by the increase in misfolded and unfolded proteins, and intended to re-establish protein homeostasis. CRT recognizes misfolded proteins and binds to them, preventing them from leaving the ER, while EIF2S1 attenuates mRNA translation, preventing an influx of misfolded and damaged proteins into the ER [43,63,64]. Enriched levels of CRT and EIF2S1 are evidence of ongoing protein repair in midgut epithelial cells following Pqt treatment. Furthermore, oxidative stress is associated with decreased levels of calcium ion (Ca^2+^) in the ER lumen, which further impairs the ER’s ability to function properly. The ER has the highest concentration of Ca^2+^ in a cell compared to the cytosol or other cellular organelles [65]. This concentration is regulated by transporter and channel molecules that are involved in the uptake or release of Ca^2+^ between the cytosol and ER lumen [66,67]. Ca-P60A is a transporter in the ER membrane that is involved in the uptake of Ca^2+^ from the cytosol into the ER lumen. Enrichment in this protein is evidence of a Ca^2+^ imbalance that is associated with ER stress and intended to increase the concentration of Ca^2+^ in the ER of midgut epithelial cells. CRT is also capable of binding to Ca^2+^, and therefore is involved in the regulation of Ca^2+^ homeostasis within the ER [68,69]. This shows that the enriched CRT levels were also involved in ensuring there is high Ca^2+^ levels in the ER of midgut epithelial cells.

A limitation of the study was that we could not directly measure by LC-MS/MS the protein profile of the midgut in response to Pqt in a blood meal. Therefore, the difference in the expected versus observed protein response profiles could be a result of the route of exposure to Pqt, i.e., ATP-saline solution versus blood meal. We observed a completely different pathway for redox homeostasis than previously described by others, which suggests that the mosquito midgut has in place at least two cellular response mechanisms that partition based on the manner by which ROS are induced in the tissue. The homeostatic regulation of ROS during mosquito blood feeding is well established [21,22,23], and it is likely that the mosquito can tolerate concentrations greater than 1 mM of Pqt due to the mitigating effects of several ROS mediation pathways that are active in concert in the midgut. One reasonable interpretation of these results in the context of our hypothesis and proposed transmission-blocking drug paradigm is that in a real transmission scenario, complete with Pqt-like drugs, a blood meal, and malaria parasites, the mosquito will likely remain unaffected by high levels of ROS, whereas the parasite will succumb.

## 5. Conclusions

We have shown that the *An. gambiae* midgut response to Pqt-mediated oxidative stress initiates an ER stress pathway rather than inducing the canonical Trx or GSH-dependent antioxidant proteins. Quite the opposite has been shown for Pqt-mediated oxidative stress in *Plasmodium* (in both asexual and sporogonic stages), where it is mainly regulated through Trx and GSH-dependent proteins [15,70]. This difference in the regulated response to Pqt between *Anopheles* and *Plasmodium* could be harnessed as an intervention strategy against *Plasmodium* development in *Anopheles* midguts. Pqt at the concentration used in our experiments is known to be effective against the parasite [15], but is not immediately harmful to the mosquito. However, the potential utility of Pqt as a transmission-blocking compound was not explored in this present study, and Pqt’s non-specific toxicity in different cell systems precludes it from such a direct translational application. Further studies are needed to screen a suite of additional Pqt-related drugs that can fulfill this role. Ideally, this screen would identify a shortlist of repurposed, druggable compounds and an appropriate, safe dose that is selectively toxic and deleterious to *Plasmodium*, but yet allows *Anopheles* and human hosts to remain unaffected. The identified drug compound(s) can then be deployed to a field setting to evaluate for its transmission-blocking effect against locally circulating parasites.

## Figures and Tables

**Table 1:** Proteins enriched in paraquat (Pqt)-treated midguts that are directly involved in mitigation of the induced oxidative stress.

Protein Description	Fold Change	*p*-Value	Function
ABCC8 (AGAP008437) ATP-binding cassette transporter (ABC transporter) family C member 8	4.37	0.015	Upregulated in bendiocarb resistance *Anopheles gambiae*, a detoxification gene [31]
ALDH6A1 (AGAP002499) Methylmalonate-semialdehyde dehydrogenase (acylating), mitochondrial	4.28	0.035	Classified as environmental and oxidative stress proteins [32]
Ca-P60A (AGAP006186) Calcium-transporting ATPase sarcoplasmic/endoplasmic reticulum type	5.99	0.039	Function impaired by oxidative stress [33,34,35,36]
CRT (AGAP004212) Calreticulin	1.64	0.017	Ca^2+^ homeostasis [37,38,39] and pro-apoptotic protein [40]
EIF2S1 (AGAP011190) Eukaryotic translation initiation factor 2 subunit alpha	1.69	0.034	Conserved in eukaryotes, the phosphorylation form of this protein serves as a signal of cell survival by attenuating the translation of mRNA [41,42,43]
ODC (AGAP011806) Ornithine decarboxylase	2.72	0.043	Upregulated after ivermectin-containing blood meals [44]
SDHB (AGAP007309) Succinate dehydrogenase (ubiquinone) iron-sulfur subunit	4.23	0.045	Ferredoxin balance system

The data is summarized into four columns. In the first column, the name of the protein is described with its abbreviated form and accession number. The second column shows the fold change in enrichment level for each of the described proteins. In the third column, *p*-value (*p* < 0.05) results of the Student’s *t*-test on the fold change in enrichment level are reported for each of the described proteins. The fourth column gives a brief summary of the function of the described protein, with any associated references.

**Table 2:** A list of Trx and glutathione (GSH)-dependent transcripts evaluated following *P. falciparum*-infected blood meal ingestion.

Transcript/Accession ID	Function/Annotation	Response to *P. Falciparum* Blood Meal Ingestion.
Thioredoxin-1 (Trx-1; AGAP009584)	Dithiol–disulfide exchange reaction with GSSG to produce GSH [12]	None (*p*-value = 0.3088)
Thioredoxin-2 (Trx-2; AGAP007201)	Antioxidative function as electron donor to TPx [49]	None (*p*-value = 0.7309)
Thioredoxin reductase (TrxR; AGAP000565)	Key enzyme of the Trx system responsible for replenishing Trx-1 [50]	None (*p*-value = 0.8806)
Thioredoxin peroxidase-1 (TPx-1; AGAP000396)	Antioxidant enzyme that catalyzes peroxides [49]	None (*p*-value = 0.7976)
Atypical 2-Cys peroxiredoxin (Peroxiredoxin V; PrxV; AGAP001325)	Antioxidant enzyme that protects against ROS/RNS [13]	None (*p*-value = 0.8736)
Glutathione synthase (GS; AGAP000534)	Involved in the GSH biosynthesis pathway	None (*p*-value = 0.8515)
Glutathione peroxidase (GPx; AGAP004247)	Antioxidant enzyme that catalyzes peroxides [14]	None (*p*-value = 0.8998)
Glutathione S-transferase delta class 1 (GSTD1; AGAP004164)	Implicated in insecticide resistance and detoxifies xenobiotic compounds [51,52]	None (*p*-value = 0.9195)
Glutaredoxin-1 (Grx-1; AGAP011107)	Essential component of the GSH system [53,54]	None (*p*-value = 0.4838)

## Supplementary Materials

The following are available online at http://www.mdpi.com/2227-7382/6/4/47/s1, Table S1: Protein identification and peptide information raw data, Table S2: Primer sequences for qRT-PCR analysis, Table S3: Proteomic data on differential expression between sugar and Pqt treatment groups, Table S4: Statistical analysis of qRT-PCR transcript data.
