# Supplementary material for: Paraquat-Mediated Oxidative Stress in Anopheles gambiae Mosquitoes Is Regulated by An Endoplasmic Reticulum (ER) Stress Response
Source: Proteomes. 2018 Nov 12;6(4):47. doi: 10.3390/proteomes6040047 (PMC6313908; doi:10.3390/proteomes6040047)
Supplement: Supplementary file 1 [file proteomes-06-00047-s001.zip › Pqt_Paper-Table_S2.doc]

**Supplementary Table 2**: Primer sequences for Trx- and GSH-dependent transcripts confirmed with qRT-PCR.

| Thioredoxin pathway related transcripts | | | |
| --- | --- | --- | --- |
| Transcript | Primer sequence | | Length (bp) |
| Thioredoxin-1 (Trx-1) | F  R | 5’ – CGTGCAGCAGCAGCAAATCG – 3’  5’ – TTACATGGTGAAAGATTCCGAGGA – 3’ | 103 |
| Thioredoxin-2 (Trx-2) | F  R | 5’ – CGGTGATCGTGGACTTCTTT – 3’  5’ – GTCCGTGTGCTCGTCTATATC – 3’ | 131 |
| Thioredoxin reductase (TrxR) | F  R | 5’ – CAGCAATGTGTGCATGTGATT – 3’  5’ – CTGTTGTTCCTTCGCTCTACTT – 3’ | 102 |
| Thioredoxin peroxidase -1 (TPx-1) | F  R | 5’ – CATATCGCAGCAAACACACAC – 3’  5’ – GGCAGAGCACTAAACGATGA – 3’ | 144 |
| Atypical 2-Cys peroxiredoxin (Prx-V) | F  R | 5’ – CTGAACGCTGCTGCTACAT – 3’  5’ – GCGGGTGAATCCTCAAACA – 3’ | 95 |
| Glutathione pathway related transcripts | | | |
| Glutaredoxin-1 (Grx-1) | F  R | 5’ – GTGGTTATCTTCTCCAAGACC – 3’  5’ – CATCGTTACGCTTGTCCA – 3’ | 109 |
| Glutathione S-transferase delta class 1 (GSTD1) | F  R | 5’ – AGCAATCGTGTTGTCTC – 3’  5’ – CGGGTAGGTAATAGAAATCC – 3’ | 107 |
| Glutathione peroxidase (GPx) | F  R | 5’ – AGAAGTACCGTGGCAAG – 3’  5’ – CACCGAACTGATTGCAG – 3’ | 150 |
| Glutathione synthase (GS) | F  R | 5’ – TGCTGGAAACAGGTGGAAA – 3’  5’ – CCAAGCTCCGACAGAATGTA – 3’ | 98 |
